# Digital twins and the digital logics of biodiversity

**DOI:** 10.1177/03063127241236809

**Published:** 2024-03-21

**Authors:** Michelle Westerlaken

**Affiliations:** University of Cambridge, Cambridge, UK

**Keywords:** biodiversity, digital twins, sensing, automation, monitoring

## Abstract

Biodiversity is a multidimensional concept that can be understood and measured in many different ways. However, the next generation of digital technologies for biodiversity monitoring currently being funded and developed fail to engage its multidimensional and relational aspects. Based on empirical data from interviews, a conference visit, online meetings, webinars, and project reports, this study articulates four digital logics that structure how biodiversity becomes monitored and understood within recent technological developments. The four digital logics illustrate how intensified practices of capturing, connecting, simulating, and computing produce particular techno-scientific formats for creating biodiversity knowledge. While ongoing projects advance technological development in areas of automation, prediction, and the creation of large-scale species databases, their developmental processes structurally limit the future of biodiversity technology. To better address the complex challenges of the global biodiversity crisis, it is crucial to develop digital technologies and practices that can engage with a wider range of perspectives and understandings of relational and multidimensional approaches to biodiversity.

The term ‘biodiversity’ only rose to prominence in scientific literature and policy in the 1980s, and was formally included in intergovernmental science policy in 1993, with the formation of the United Nations Convention of Biological Diversity (CBD) (CBD, 2022; Díaz & Malhi, 2022). Within this policy, ‘biodiversity’ initially referred exclusively to the number of different species. However, recent frameworks for biodiversity have become multidimensional, examining diversity in relation to the richness of organisms, their distribution, biomass, metabolic activity, (phylo)genetic difference, or other species compositions within entangled ecosystems (Díaz & Malhi, 2022). Less techno-scientific conceptions of ecosystems understand biodiversity not only through resource use, but through social and cultural values, long-term detailed observations of subtle changes, and multispecies lifeways and experiences (Heiner et al., 2019; Moller et al., 2004; Moorcroft et al., 2012; Verran & Christie, 2007). Historically, the concept and popularity of biodiversity in Western science arose from an entanglement of philosophical, personal, political, cultural, and empirical values and beliefs that are essential to understanding contemporary conservation projects (Heise, 2016; Sepkoski, 2020). ‘Biodiversity’ is a contested concept, meaning different things to different people and in different contexts. As a result, the monitoring of biodiversity changes depending on the values and variables included.

Today, measuring and monitoring biodiversity through increasingly digitized environments offers the potential to generate insights that can inform policy and decision-making. The funding dedicated to recent technological advances shows that governments, scientific institutions, and commercial entities have high hopes that new digital technologies can further these aims. At the same time, techno-scientific practices of producing biodiversity knowledge are being criticized for their fragmented, reductionist, distancing, and economic orientations. This article summarizes and contributes to this critique, arguing that new technological developments do not necessarily contribute to increased biodiversity or ecosystem restoration. Over the next decade, many new technologies for monitoring biodiversity will likely emerge that intensify practices of automation, prediction, and sensing, contributing to large-scale infrastructures for generating biodiversity knowledge. These developments include technologies such as digital twins and their connected sensors and infrastructures.

A ‘digital twin’ is a network of different data sources that is treated as a real-time digital copy of a physical entity. Recent social science scholarship on the digital twin phenomena is rapidly expanding, often focusing on the ontological consequences and limitations of the digital or virtual ‘double’. This echoes older work in fields such as virtual worlds research and game studies where digital doubles, avatars, and subjectivities have been studied for decades (Crang et al., 1999; De Mul, 2010; Grimshaw, 2013). Rather than understand digital twins for biodiversity as a process of doubling, this article explores how they shape a particular understanding of biodiversity, through digital logics that have large-scale ramifications for environmental futures. The study asks how the modelling of biodiversity data in digital simulations furthers reductionist and fragmented modes of understanding that risk erasing more complex and relational knowledge of living entities in ecosystems. Ultimately, the goal is to question the design- and data-generating decisions that install limited digital simulations, in order to move towards more multidimensional modes of digitalizing biodiversity.

## Open data, online conversations, and field notes

This article is informed by empirical materials from interviews, webinar data, and field notes of a conference at a project site. Between November 2021 and May 2022, our research group interviewed 33 people, from around the world, whose work and/or livelihoods engage with environments and digital technologies. These people include industry professionals, governmental actors, community organizers, Indigenous activists and conservationists, academics, and industry researchers. Starting from this broad focus area, a narrower focus on biodiversity monitoring and digital twin technologies emerged through these conversations, which subsequently guided follow-up interviews and research. All 33 interview recordings were transcribed, translated to English when undertaken in a different language, and coded through qualitative data analysis with Atlas.ti software. With permission of the participants, most interviews have been edited as abbreviated podcasts and made publicly available through the Smart Forests Atlas, an open data platform available via https://atlas.smartforests.net/

As part of a wider case study into digital twins and its connected sensors and infrastructures, this study also includes data found through analysis of over 30 hours of online webinars and meetings. However, as will become clear, these materials risk narrowing the data to initiatives by groups that benefit from sharing their work online, such as industry professionals and academics. Furthermore, despite interviews being carried out with participants across the planet, the data in this article show that conversations around the next generation of biodiversity technology predominantly take place through funded research projects and industry initiatives in the Global North, with publicly funded projects specifically occurring in Northwestern Europe. The fourth digital logic (on computing) further reflects on the potential inequalities that arise from this limited participation in the development of new biodiversity technology.

## The challenges of understanding biodiversity

Measuring biodiversity usually involves documenting species and working with incomplete datasets and aspects of ecosystems that lie beyond available knowledge. Most species, especially bacteria, fungi, and eukaryotic species (such as algae and moulds), are minimally documented and understood. Scientists emphasize that biodiversity metrics are high-risk, imperfect, and may involve high uncertainty due to unknown factors that can trigger nonlinear, unforeseen, or irreversible changes (Steffen et al., 2015). Contrary to climate change data, which has an internationally agreed baseline referring back to preindustrial times, there is no baseline for measuring historical biodiversity loss (Brörken et al., 2022; Donadio Linares, 2022). Since the meaning of biodiversity continues to change, methods for its mapping are controversial, contested, and enact different realities and scientific ideals (Aspøy & Stokland, 2022).

Besides inevitable inaccuracies, measuring biodiversity only through abundance and extinction rates excludes more comprehensive knowledge on how organisms affect ecosystems. Multidimensional studies show, for example, that biodiversity loss affects certain organisms over others, and that some organisms even thrive in response to landscape degradation (Tsing, 2017). To take another example, the impact of losing slow-growing and long-living megabiota such as large trees can have larger effects on local ecosystems than the loss of other organisms (Díaz & Malhi, 2022; Simard, 2017). Moreover, from the broader field of development studies, biodiversity monitoring is interrogated for its lack of engagement with global inequalities, local values, and social justice (Biermann & Kim, 2020). Authors from different disciplines argue that global and local inequalities and differences must be incorporated into any scientific framing that suggests (or informs) decision-making on environmental issues (Biermann & Kim, 2020; Dao et al., 2018; Leach et al., 2013; Raworth, 2014; Schmidt, 2013). This critique has been further elaborated within Science and Technology Studies (STS), by questioning who decides the precise values of ecosystem processes, and how they are translated to actual governance (Biermann & Kim, 2020; Pickering & Persson, 2020). When scientists alone determine which rates of biodiversity loss are dangerous and how these should be measured, the responsibility and political power may shift from policymakers to scientific experts, and policymakers may be tasked only with ensuring that boundaries are not transgressed (Biermann & Kim, 2020).

Besides critique of the reductionist scientific approach to monitoring biodiversity (see also Bowker, 2000, 2007), STS researchers have articulated how such monitoring has become embedded in an economic system in which biodiversity rates can be valued and exchanged. Over the past two decades, expressions of biodiversity as quantifiable, fragmented parts have become liable to mapping, counting, and economic trade (Turnhout et al., 2013). The economic logic of valuing biodiversity as an ecosystem service has been criticized by STS scholars (Büscher et al., 2012; Ellis et al., 2010; Turnhout et al., 2013; Yusoff, 2010). For example, Turnhout et al. (2013) warn of the risk that the single measure that expresses biodiversity will substitute for much more complex ecosystems, whereby the measure itself—and not what it represents—becomes what is valued. Such reflections also connect biodiversity monitoring techniques with earlier STS work on how measurements enact new realities and are intrinsically political (Latour & Woolgar, 1979; Stengers, 2000). The scientific measurement of biodiversity produces relevant knowledge, but it obscures more situated and complex understandings of ecosystems. As a result, biodiversity conservation becomes focused on meeting numerical targets—including by identifying as many new species as possible—establishing extinction rates, or maintaining a singular biodiversity threshold. Large funding schemes are being directed towards these goals in the hope that monitoring and measuring will ultimately contribute to preventing biodiversity loss and restoring ecosystems.

The reductionist scientific logics of biodiversity monitoring and the economic logics of valuing biodiversity as ecosystem services reinforce each other in establishing an order within which the fragmented quantifiable knowledge and value of biodiversity are embedded in society. Over the coming decades, digital technologies will further define this order through increasingly powerful processes of automation, prediction, and simulation. The most recent developments in biodiversity monitoring include technologies that will be used to direct and manage biodiversity through digital environments, including digital twins and their connected, large-scale, digital infrastructures. This article demonstrates that these developments not only perpetuate reductionist approaches, but reinforce biodiversity as a singular entity.

## Biodiversity, digital twins, and emerging digital platforms

The concept of a digital twin has been around for decades, but due to increased computational power such applications are becoming more widespread, especially in manufacturing and construction industries (The National Digital Twin Programme, 2023). A digital twin can be defined as a network of different data streams deriving from sensor technologies and other types of observations to create a real-time digital copy of a physical entity. These digital environments can then be used to simulate and predict events, and can even directly interact with their physical counterparts. With the rise in popularity of digital twin applications, new developments also focus on twinning more complex ecosystems, such as forests (Buonocore et al., 2022; X. Jiang et al., 2022; Nita, 2021; Sanchez-Guzman et al., 2022), human bodies and organs (Bruynseels et al., 2018; Erol et al., 2020), oceans and coastlines (Barbie et al., 2022; P. Jiang et al., 2021), islands (Cressey, 2015; Peters, 2022; Shepherd, 2022), and species (Lobato-Rios et al., 2022). Within these and other environmental technology projects, ‘digital twins’ is used as a buzzword, and the projects focus on small subsets of data types to generate digital simulations. For example, a ‘digital twin’ of a forest typically focuses on simulating carbon-offsetting or tree-planting data (Silva et al., 2023). This study therefore situates digital twinning with the emerging connected technologies that produce the source data for these projects. The open-data and open-source algorithms of new biodiversity monitoring technologies already provide input for the creation of digital twins. This is particularly clear in the EU-funded Destination Earth project.

Destination Earth—or DestinE—is a large European Space Agency project that aims to create a highly accurate real-time digital simulation of our planet to understand and monitor the effects of natural and human activity, anticipate extreme events, produce actionable predictions, and inform policies on climate-related challenges (ESA, 2022; Nativi et al., 2021). Since the launch of Earth observation Landsat satellites in the 1970s and the popularization of the ‘Digital Earth’ metaphor to digitally model Earth systems in the late 1990s, many digital technology initiatives to create Earth replicas have emerged (Barton, 2023; Guo et al., 2020). These programs and technologies have changed scientific approaches in fields such as ecology and interdisciplinary Earth sciences (Kwa, 2005). Now, in this new ambitious project, Destination Earth promises to integrate Digital Earth replicas with different domains (one of which is described as ‘biodiversity’) and form ‘one comprehensive digital twin of the complete Earth system’ (ESA, 2022). The platform is planned for full launch by 2030 and has received €150 million initial funding from the European Commission for its first phase from 2021 to 2024 (Bauer et al., 2022). Most of the publicly funded European participants and projects that were part of this study do not directly work for Destination Earth, but they repeatedly describe aiming towards integrating their biodiversity data with this initiative (detailed below). A variety of other next-generation biodiversity digital twins and connected infrastructures are currently being developed worldwide. Commercial entities such as Microsoft’s ‘AI for Earth’ program and Microsoft Azure software are building a ‘Planetary Computer’ with petabytes of environmental monitoring data (Microsoft, 2021, 2022). Another large commercial player involved in developing next-generation biodiversity monitoring systems is the Nvidia Omniverse Platform, which provides cloud computing and developmental software required to work with the large amounts of data that digital twin systems need to function (NVIDIA Omniverse, 2022). Geographic Information System (GIS) company ESRI is also used for a variety of geospatial digital twin applications to map and model biodiversity (ESRI, 2019; Peters, 2022; Wright & Perkl, 2022). Besides commercial entities, urban governmental initiatives are expanding their ‘smart cities’ development with digital twins by including environmental data, such as Singapore’s GeoSpace-Sea, which brings together different real-time data sources on land, marine and coastal environments (Begum, 2021).

Many publicly funded research projects have emerged in the European Union, focused on advancing biodiversity technology for digital twins into areas like automating species recognition, expanding DNA barcoding, and connecting data infrastructures. For example, the German project Automated Multisensor Stations of Monitoring of BioDiversity (AMMOD) works with sensor technologies such as radars, camera traps, acoustic recording, and odor detectors with the aim of automating species recognition to identify and document species and create a large-scale integrated biodiversity data network (AMMOD, 2022). The Dutch project ARISE was allocated €18 million in initial funding from the Dutch government to build a digital infrastructure to ‘identify and monitor all multicellular species in the Netherlands’ (ARISE, 2022a). This project aims to create a rapid species detection platform that can tell which species are present from any type of sample, whether an image, DNA sample, or sound recording (ARISE, 2022b). The EU-funded MAMBO project currently aims to develop knowledge, tools, and infrastructure for automating biodiversity monitoring through sensor development, deep learning, computer vision, acoustics, remote sensing, citizen science, data pipelines, and ecological modelling (MAMBO, 2023). The research project BioDT, also EU-funded, stands for Biodiversity Digital Twin, and exploits the recently launched Finnish supercomputer LUMI to develop a prototype that can provide advance models for biodiversity simulation and prediction (BioDT, 2022). The Greek EU-funded project Iliad aims to build a digital twin of ocean and marine environments, integrating existing EU earth observations and digital infrastructures (Iliad, 2023). All of these ongoing projects aim to create EU-wide biodiversity monitoring systems, many of which directly articulate the aim of becoming interoperable with Destination Earth. Lastly, during the 2022 biodiversity COP15 in Montreal, The Group on Earth Observations Biodiversity Observation Network (GEO BON) presented the need for a global biodiversity observation system (GBiOS), a federated network for monitoring biodiversity across the planet in order to combine technological advances and databases (GEO BON, 2022).

These initiatives share an ambition to use new computational technologies to create large-scale digital infrastructures that combine different and real-time data sources to measure and monitor biodiversity. Instead of interactive digital platforms that present singular data sources such as image or satellite data next to one another, these projects promise to develop infrastructures that can incorporate and combine different formats of biodiversity data that can be read by algorithms to present predictions, simulations, or automated species identification. A digital twin, in this context, comprises a digital copy, representation, or simulation of a physical environment that can be used to monitor and mirror ecosystems in real-time, predict its dynamics, or even directly intervene in ecosystems (Datta, 2017; Korenhof et al., 2021; Luan et al., 2021).

The use of digital technologies for measuring, monitoring, and simulating biodiversity has already been studied by STS scholars for several decades. Their critical reflections can be extended to the intensification of digital efforts in the form of digital twin technologies. Already in 2000, Bowker argued that datafication actively reshapes our understanding of biodiversity (Bowker, 2000). Environmental digital technologies generate distinct ways of ‘tuning in’ to environments: They value distinct environmental features, and they inform decisions and practices about how to manage and govern environments (Gabrys, 2016). Choices about which biodiversity data to prioritize, assumptions made in the analysis, simplification of more complex biodiversity dynamics, and exclusion of other forms of knowledge about nonhuman life risk creating injustices in the ways organisms and environments are represented and policy decisions are made (Gabrys et al., 2022; Pritchard et al., 2022). For example, when emerging digital technologies for biodiversity monitoring are mostly developed and funded in the Global North, inequities arise regarding how these technologies are designed, and who does the labour involved in gathering data in biodiversity hotspots—that are often located in the Global South. Knowledge production thereby becomes redistributed on a large scale, which affects who creates and owns data (Heaton, 2022; Leonelli, 2015; Nadim, 2016). This not only raises questions regarding authorship and accountability, but increases the distance between data generation and the ultimate goals of biodiversity loss prevention or ecosystem restoration. While digitalization may increase knowledge of organisms and their habitats, its transformative potential is often assumed but far from guaranteed. Sheikh et al. (2023) furthermore point out that biodiversity is increasingly perceived and governed via digital technologies that rarely account for limits to human perception and thereby reinforce a type of human exceptionalism where multispecies agencies become invisible in digital environments. Finally, increased computational complexity requires considerable energy resources, including physical servers, datacentres, and information networks that can create more, rather than less, biodiversity loss simply through their energy use (LeBel, 2012; Nost & Goldstein, 2022).

## Capturing: Digital sensor capacities define species monitoring

The technological capacities of the different sensors used and developed for monitoring biodiversity are actively shaping which species are included and excluded from data, policy, and conservation. Many of these sensors have been around for decades, but they are now being developed for automating species recognition. Previously, terabytes of camera trap images, acoustic recordings, and metadata had to be manually checked to identify species presence in each individual file. Over the next decade, as image and audio recognition software evolves, identification will become increasingly automated. The sensors send data directly to the cloud, files are analysed by an algorithm, metadata (such as time and place of recording) are automatically added, and files are sorted to present human users with the results they may find relevant.

In our conversations with the people currently building these technologies, it became clear that decisions about which organisms to include in these automation practices are not driven by the importance of understanding certain species, nor by biodiversity policy. Instead, technologists are deciding which species to include or exclude for automated identification by the ease with which sensors and algorithms can pick up their identity and presence. A computer scientist in the UK explains how bats are one of the most analysed taxa through acoustics, because they are easier to sense:

Bats’ [acoustic data] are very mature because it’s relatively easy to detect bats. You could automatically detect a bat, computationally, because there’s very little in the background, there’s very little that sings at those frequencies. And so certainly bat surveys are probably the front runners in terms of the technology because they’ve had algorithms that can automatically spot the bats from recordings for a long time. … it’s not clear that the importance of species is correlated with how easy they are to detect. … It’s probably equally important in the UK to monitor particular bird species but it’s much harder to go out and do that. So, a lot of the focus has necessarily been on, well, things we can actually sense. (Computer Scientist, UK)

Some participants argue that focal species can be used to develop a technology, which can then be expanded to include more species. By prioritizing the technological development over a previously identified need for specific biodiversity data, such approaches risk prioritizing innovation for its own sake. What becomes invisible in such technological development cycles is how successful sensing for specific use cases affects subsequent decisions and innovations. The algorithms that identify bats tune into high frequencies and can then be used to detect other species making sounds at that range. The company responsible for the bat sensing, which has one of the largest acoustic monitoring product initiatives globally, initiated acoustic sensing technology projects for cicadas, organisms that similarly express themselves at high frequencies. The company’s technological developments drive biodiversity monitoring in certain focal areas and not others. The resulting data subsequently shapes our understanding of biodiversity and informs policy in particular directions (Borgelt et al., 2022). Automation technologies can thus become tools for leveraging political or financial power, where selective measurement shapes environmental management (Cusworth et al., 2023; Möllers, 2017). It is crucial to address how knowledge and policy decisions thereby become driven by technological capacities, and not the other way around.

This phenomenon also emerges in the case of automating insect monitoring through camera trapping. An interviewed insect-camera trap developer in the Netherlands explains that insect monitoring with their system takes place only between March and September, because the available sunlight in those months enables sufficient solar charging for their system. They further explain that training the algorithm to automatically identify an organism requires a minimum of about 50 different pictures of that particular species. Thus, only insects appearing on camera with this frequency or higher are included in the automation software. Further, the developer emphasizes the risk of exclusion by detailing how the system focuses only on flying insects that are attracted to the colour yellow:

A risk is that you only attract specific types of insects and not everything you want to see. So you don’t get a complete overview. For example, we know that pollinators are not really attracted to the [yellow] screen we use at the moment, so you don’t see them. … And you can only collect flying insects at the moment with this one. So, if you want to have ground insects you need to find a different version or a different way to collect them. (Camera trap developer, Netherlands)

On multiple occasions we learned from our interviewees that the decision to focus on sensing and automatically identifying certain species is often informed by technological possibilities and limitations, and not by a search for the most relevant biodiversity data. Once such development directions become integrated into the sensor hardware, detection algorithms, or digital infrastructures, they reinforce innovation and data accumulation towards certain species and not others. Large-scale digital twin technologies then aim to predict biodiversity rates with calculations based on this very partial sensor data.

Recently, researchers have emphasized the importance of increasing our understanding of species that have become systematically excluded from sensing practices, such as soil organisms, fungi, and amphibians (Borgelt et al., 2022; Pritchard et al., 2022). Besides involving different types of species in the development of new sensor technology, it is also crucial to establish more interdisciplinary teams where expertise in technology, ecology, policy, design, and other relevant fields can rethink technological decisions regarding how focus areas are determined, who directs technological innovation, and how funding is allocated towards certain innovations over others. More specifically, this analysis shows how wider expertise must become involved earlier on in decision-making and funding processes.

## Connecting: Digital infrastructures that complicate project development

We don’t know exactly what it will look like, we don’t know what the future will bring. We kind of know what we want to do this year. We have done a few pilots for instance to figure out, is this the direction we want to take, or is that the technology that we want to use. And we’re going from those pilot phases into a scale up phase. (Programme lead, Netherlands)

The technological complexity and large-scale connected digital infrastructures required to build biodiversity digital twins means that many projects begin by defining, prototyping, and testing smaller pilot scenarios, with the aim of later scaling up. Terms such as ‘agile design’, ‘hackathons’, ‘sprints’, and ‘proof-of-concept’ regularly surface in these projects. These developmental methods help to break down larger projects with unknown final outcomes into smaller, more manageable, and iterative challenges, with frequent deadlines that can be completed in individual project phases or teams. Such pilots can focus on a single species or taxa (e.g., bats) or involve a distinct subset of end-users (e.g., ecologists), project phase (e.g., data collection), ecosystem events (e.g., hurricanes), local regions (e.g., a nature reserve), data types (e.g., DNA barcodes), sensors (e.g., automated camera traps), or algorithmic tasks (e.g., species identification). Yet the articulated visions—such as creating digital twins, building full digital replicas, becoming a one-stop shop for all biodiversity data, or automating the mapping and identifying of all species from any type of sample—are much larger than any combination of ongoing pilot projects.

Documentation on the Destination Earth project by the European Space Agency outlines that a digital twin of the Earth must be flexible and dynamic because it operates in an environment in which technology, policies, and user needs are in constant evolution (Nativi et al., 2021). The authors argue for a technological architecture that is highly flexible, modular, scalable, and independent from any specific provider, technology, or license. However, several ongoing biodiversity technology projects clarify the contrasts and disparities between current pilot solutions and such overarching visions:

We have teamed up with [e-science institution], who will do a one-week hackathon to build the first prototype of this [DNA barcode] database. (DNA sequencing project lead, Netherlands)

We started with prototypes that were working for small use-cases. … But it kind of turned out that that was not quite sufficient enough for the variety of data types that we are planning for. (Data management project lead, Netherlands)

The third objective is to ensure interoperability with these other digital twin initiatives. One example is Destination Earth, and we hope to set up these showcases to look at how to synchronize the [project] digital twins that we will develop with other digital twins. So, this means we need to work in unison with the other DT projects that are happening and will happen also in the future. (Principal investigator, Finland)

And it if works on Moorea [a remote island in French Polynesia], the approach could be rolled out to other parts of the world. (Davies in Cressey, 2015, p. 256)

These prototypes, pilots, and showcases are essential to better understanding different scenarios and working iteratively towards larger project goals. However, they may have significant limitations. Can a one-week hackathon by an external party establish very partial solutions for a large-scale digital infrastructure that then becomes embedded in the project? The hackathon’s goal of creating a prototype within a limited time forces developers to simplify features and narrow use cases (Irani, 2015).

In the third quote just above, the project manager of a recently launched biodiversity digital twin project describes how interoperability and synchronization with other environmental digital twins will be ensured. It is relevant to note here that this project consists of a three-year, EU-funded research initiative that lists 22 partners and eight use cases. This raises crucial questions regarding the project’s potential to achieve interoperability with large-scale European infrastructures that are still in early development stages. The term ‘showcases’ here suggests a much narrower approach in which the outcomes include examples of possible connections with other infrastructures, rather than actual interoperability.

In the above example concerning Moorea—one of the most studied ecosystems in the world, thanks to its remote location and small size—an ongoing digital twin project received $5 million in funding under the assumption that its outcome can be ‘rolled out’ elsewhere in the future. Yet, there are vast differences between remote ecosystems with long-established data infrastructures and other regions where shared national borders and disconnected data and technology on biodiversity must be brought together to create interoperable digital infrastructures. While this project can indeed inform future digital twin initiatives, the expectation that it can simply be ‘rolled out’ elsewhere ignores the long-standing challenges and conflicts in bringing together national and international infrastructures, policies, and biodiversity knowledge (Gugganig, 2021; Hennessy, 2018; Laurent et al., 2021). Such differences require fundamentally different approaches.

The plethora of pilots, prototypes, and use-cases that aim to advance digital technology suggests that such developmental approaches may be favoured by funding bodies because they have clearly identifiable outcomes and timescales (Edwards et al., 2007). The short-term funding structures of these projects contradict the longer timescales on which biodiversity digital twins are expected to operate. By establishing short-term objectives and narrowing down features, it becomes possible to develop solutions for a specific scenario. Yet such short-term solutions can actually contradict more ambitious project goals. Instead of helping to consolidate digital biodiversity technology into a unified infrastructure, these projects result in relatively discrete pilots and case studies.

Is a unified infrastructure possible, desirable, or needed? The ambition to create overarching technological systems and unified digital replicas or simulations is an extension of a long-standing modernist goal of creating simple understandings of complex phenomena. On the one hand, biodiversity digital twin ambitions articulate the objective of creating an overarching replica of enormously complex ecosystems. On the other hand, the numerous disconnected pilots demonstrate that understanding biodiversity requires a case-by-case approach. Therefore a more plural tapestry of patchwork programs may be more helpful in producing multidimensional biodiversity knowledge, using different technologies and relying on a variety of data infrastructures, without claiming completeness. The complexity of ecosystems and the relational dynamics between species can hardly be expressed in a digital system built by humans. Addressing this issue requires rethinking how digital technologies can contribute to a pluralistic understanding of biodiversity. Scaling up digital technologies therefore involves asking challenging questions about how algorithms can help to produce—rather than eliminate—plurality and how connected digital infrastructures can encourage users to engage with data in ways that acknowledge that they generate multidimensional insights.

## Simulating: Fragmenting measurable entities into coherent digital replicas

Many current technological innovations on biodiversity focus on automating species identification, but questions of biodiversity can concern a much wider variety of processes within different ecosystems. For example, biodiversity—or the lack thereof—can be related to the health of different organisms and their habitats, the relations between different species, their behaviour, or changes within ecosystems at large. Recently, multidimensional approaches have begun to explore how ecosystems are entangled and how relations between species provide important knowledge on ecosystem degradation (CBD, 2018; Forest Peoples Programme, 2020). These approaches help to rethink the exclusive focus on genetic diversity by incorporating other kinds of diversities (van Dooren, 2009; Puig de la Bellacasa, 2015). Beyond Western scientific knowledge on biodiversity indicators, Indigenous communities have understood the value of biodiversity not only through resource-use but also through the social and cultural values of their territories (Heiner et al., 2019; Moorcroft et al., 2012). Furthermore, lived experience and harvesting/hunting practices of local communities help to understand subtle environmental processes by identifying changes in biodiversity through detailed and long-term observations (Moller et al., 2004). Digital technologies are sometimes used to tell the stories of Indigenous lands and people and preserve its ancestral importance, whereby biodiversity data does not just represent environmental processes but actively performs and makes them (Verran & Christie, 2007).

However, the digital technologies that are currently developed in the Global North do not seem to consider biodiversity as a multidimensional issue that involves many forms of knowledge, but predominantly focus on calculating biodiversity through the absence or presence of certain identifiable species. The Global Biodiversity Information Facility (GBIF), for example, is one of the largest species databases globally, and contains the reference database for many ongoing biodiversity technologies. It provides species information through static categories such as species occurrence and should be understood as only a starting point or simplified format for generating biodiversity knowledge (Slota & Bowker, 2015).

One of the ways in which digital technologies and databases work towards reducing the meaning of biodiversity to a set of identifiable elements is through the expansion and automation of DNA barcoding projects (see Ellis et al., 2010; Waterton et al., 2014). Such projects aim to catalogue DNA samples for as many different species as possible. Several ongoing biodiversity initiatives and start-ups now aim to detect any type of species that is present in a certain area. With these technologies, it is possible to collect a scoop of soil or a cup of water in a local area and identify the different species—fungi, bacteria, or DNA traces from larger species—that are present in these samples. This technology helps to understand the diversity of species that cannot currently be identified through other types of sensors and so diversifies biodiversity knowledge. At the same time, such technologies reinforce a mode of understanding biodiversity that takes place exclusively through completing a predefined target list of identifiable species:

having the list of all the species minus the list of species that we already have produces a target list, which is now publicly available … From this [digital list] we can easily export tables to give to the people that collect specimens [to say:] This is the list of species that we would like you to bring into the system for us. (DNA sequencing project lead, Netherlands)

Such an infrastructural decision helps to identify the presence of a limited list of species, but eliminates the possibility for any biodiversity monitoring beyond the presence and absence of these species. That said, a recurring argument for a focus on species is that this process can eventually be used with future machine-learning technologies that will enable more complex knowledge of biodiversity. Such arguments illustrate how new technologies to monitor biodiversity give rise to a digital logic that outsources multispecies complexity to algorithms that rely on future innovation that must fit within the database structures that are currently installed.

The data involved in simulating biodiversity in digital twins is generated by sensors, observations, genetic information, and metadata. These are all gathered in a ‘data lake’ on a central hub or platform, and become available for machine learning applications, Application Programming Interfaces (APIs), and visual representations through which users can access and combine the different data sources. Advanced computational power further enable local, private, and synchronized data processing. This means, for instance, that sensors themselves can perform more complex tasks, such as comparing sensor recordings with cloud databases to automate species identification, or sending data to other devices to minimize human involvement. With edge-computing, users can also run more data-intensive algorithms faster on their own computers, enabling access to big data to a wider variety of potential users.

Ultimately, the development of digital twins and their connected infrastructures limit themselves to entities and processes that are already identified by the humans who are building the simulated environment. The information is gathered through fragmented data streams that are subsequently brought together again in a digital system. This process first separates ecosystem entities into different data forms and then digitally recombines them with the help of algorithms that can compute large amounts of data. Nearly all participants in this study, as well as the shared perspectives in webinars and the conference, adhere to the idea that more data and more technology will produce more biodiversity knowledge. Moreover, it is generally assumed that this will directly contribute to biodiversity restoration. Nonetheless, a few interview participants reflected on the limits inherent to this ambition:

Sensors only tell part of the story—they can never tell everything because there are so many things that can go wrong—the temperature that day or the leaves that fall on top of the thing and you believe the sensor is giving you this kind of information but it’s just a leaf covering it, or something. … Mediation brings you just that far, but there’s also some other things that are just not possible to overcome with it. (Design Researcher, Finland/Colombia)

Sometimes it is … hard to make sense of all this data. So you can find yourself like lost in a world of algorithms and technology and losing a bit the sense of what am I really trying to answer here. (Remote Sensing Researcher, Canada)

If you haven’t quite checked the input data that went into that model, we say garbage in, garbage out. The outcome of that model might also not be very valid. … And models are always still a representation of the real world but it’s not always true. (Data management project lead, Netherlands)

As these participants tell us, the use of biodiversity data for algorithmic practices involves inaccuracies and uncertainties that cannot be fully overcome, given the complexity of the ecosystems they represent. The process of obtaining data from a variety of sources and combining these in a digital environment, no matter how advanced, always recreates physical ecosystems only through their measured elements (Heaton, 2022). Today, computational practices are used to manage growing amounts of data, and algorithms are replacing human predictions with automated calculations (Braverman, 2017). Such processes fail to account for unexpected relations between entities or processes that cannot be measured or predicted via algorithms. Recent innovations thereby risk seeking to validate their own biodiversity data, where digital twin systems aim to simulate larger patterns that confirm how large-scale data from different sources fit together to create a coherent digital environment (Korenhof et al., 2021). Within this mediated environment, biodiversity has to be represented as a logical assemblage where different data points fit together. This risks being unable to identify how individual outliers, counterfactual data, or processes that do not fit into this coherent system contain relevant biodiversity knowledge. It is crucial to ask what aspects are important for understanding biodiversity but are systematically excluded within these new technological developments. What does the combination of data and algorithms in digital twins fail to notice?

## Computing: Complex digital technologies that limit participation

Increasing technological complexity and the pace at which such innovation takes place limit how different stakeholders can be involved in its development. Many of the projects investigated as part of this study include proposals to involve other people in co-developing new technologies or collecting and annotating biodiversity data. Hackathons were discussed above as one of the strategies through which pilot projects are undertaken, but these are also part of a wider phenomenon that enables participation only though limited structures. While the GBIF database is regarded as a global source for species data, it predominantly contains terrestrial data from the Global North (Slota & Bowker, 2015). Expanding this data may require intensive labour from people elsewhere. During the 2022 COP15 biodiversity summit, GEO BON proposed the creation of a Global Biodiversity Observing System (GBiOS) to monitor biodiversity and inform investments through an expansive global infrastructure. They argued that mobilizing new technologies such as eDNA, acoustics, and camera traps can enable Indigenous peoples and local communities to gather data for such large-scale platforms through rapid assessments and surveys (GEO BON, 2022).

Collaborations and projects led by Indigenous and local communities can lead to important new insights on how technologies and infrastructures can be used differently to increase or pluralize biodiversity knowledge. However, such collaborations are often set out to invite local people to merely feed more data into global and national monitoring infrastructures. Rather than being collaborators with decision-making power, people are used as data sources. By collecting and logging data in digital platforms, people become data operators, which does not enable them to meaningfully interfere in changing the system (Gabrys, 2014). Such data collection methods raise questions on how environmental observations and data travels, and how trust is created between stakeholders (Kasperowski & Hagen, 2022). By streamlining all local biodiversity knowledge into one global digital infrastructure, biodiversity patterns may be uncovered, but local communities may have even fewer opportunities to structurally influence the ways in which biodiversity data is collected, stored, shared, and calculated. When such data moves between sensors, databases, countries, institutions, and individuals, it is transformed to comply with digital infrastructures that adhere to predetermined values (Walford, 2012). Funding sources, advanced computational power, data storage facilities, and technological expertise are predominantly located in the Global North and thereby ensure that environmental digital technologies and infrastructures are exclusively developed according to dominant principles and economic interests.

Further expanding this discussion on limited participation into the realm of digital twin technologies, interview participants recurringly emphasized that the projects in which they are involved are based around principles of open data and open-source software. This would enable users to apply their own algorithms, hack sensors to experiment with new monitoring practices, or otherwise reinvent how biodiversity data can be beneficial for different purposes. Yet, whether or not this really happens is highly dependent on who the users of these increasingly complex technologies are, what computational skills they have, and what their objectives in utilizing biodiversity data or technology are. In interrogating how people creatively used an open-source acoustic monitoring device, the developer shares that the people deploying these sensors are thus far not really engaging with its open-source functionality:

We thought that people would be more amenable to hacking the hardware and the software, but it doesn’t happen as much as you’d expect, because I think most of the users want to use it as a cheap device. (Computer Scientist, UK)

This reflection should encourage reconsidering ambitious aims of open-data and open-source biodiversity technology and digital twin APIs. When participatory functionality is not specifically encouraged or aligned with the technological skills that typical users have, such features risk becoming meaningless.

A related limitation for participation in emerging technologies involves the pace at which digital technologies are advanced. One participant reflected specifically on the difficulty of keeping up with commercial initiatives that use blockchain infrastructures and cryptocurrencies to value and exchange environmental entities:

I find it almost unbearable, literally, almost unbearable because I have the sense that I am permanently outpaced, permanently outpaced. It’s like two months later and there is a presentation of what is the new project or whatever, and I’m like, uh, what? What, this is going to happen? Oh, no, we haven’t thought that through. Oh, my goodness. (Director of Research at biodiversity funding initiative, Netherlands/Germany)

Critique and thoughtful experimentation cannot keep up with the pace at which digital technologies are being developed. Critical reflections emerge only after digital innovations are already installed or new projects are funded.

Over the last decade, many researchers have articulated concerns with regards to how participatory ambitions in biodiversity monitoring and other environmental technologies have reinforced existing power dynamics rather than enabled democratic involvement of different stakeholders (Paloniemi et al., 2015; Rauschmayer et al., 2009; Westerlaken et al., 2023). Several of the projects that were investigated in this study also demonstrate how participation is becoming an increasingly important aspect of biodiversity monitoring, because participatory ambitions are repeatedly highlighted in interviews, virtual meetings, and project documentation. However, within the development of increasingly complex digital twins, these participatory ambitions are often used only rhetorically and fail to be of consequence in practice. Thus, the issues that give rise to unequal participation are not resolved in these projects, but are instead becoming further entrenched in future biodiversity monitoring technologies. Ambitions to develop large-scale connected infrastructures for biodiversity monitoring are restricting rather than creating possibilities for meaningful democratic approaches due to the overarching structures they seek to impose. These findings require a closer inspection of how participatory dimensions can still be implemented in digital twin technologies and their connected digital infrastructures.

## Conclusions

The intensification of digital processes in biodiversity monitoring technologies is establishing a new generation of digital practices. Digital twins and their connected digital infrastructures combine different datasets, algorithms, and computational power to rapidly produce biodiversity knowledge. However, biodiversity can be understood and measured in more multidimensional, relational, and conflicting ways. Due to a limited knowledge of different species and a lack of understanding of the complex workings of ecosystems, biodiversity monitoring consists of partial data and incomplete environmental observations. This article summarizes findings from prior research showing that biodiversity monitoring within Western scientific and economic systems tends to simplify biodiversity into singular metrics and values, rather than attempting to encompass its multi-dimensional and relational aspects. With empirical data from interviews, virtual meetings, a conference visit, and project documentation, this study shows how such approaches to biodiversity are further embedded in new technological innovations. In these ongoing projects, predominantly financed through funding bodies in Northwest Europe, there is an emerging set of digital logics through which increasingly automated digital developments further direct how biodiversity will be understood and measured. Four such logics are identified here.

First, new automation and prediction initiatives are not necessarily striving to implement policy or articulate how biodiversity can be protected or restored. However, these digital practices themselves often guide and structure how biodiversity monitoring will take place. Technologists and developers concentrate on improving the monitoring of those species that their sensors can capture. Rather than twinning, this is a process of selective modelling based on technical capacities. These highly partial models are likely to affect subsequent biodiversity research and policy decisions.

Second, project managers and participants present highly ambitious aims to create integrated global digital infrastructures, assuming that a uniform approach towards biodiversity knowledge is possible. In practice, these digital infrastructures are funded as a series of case studies, pilot projects, prototypes, hackathons, and other separated or outsourced segments that merely offer innovative showcases. These short-term projects raise questions about the conflict between multidimensional biodiversity knowledge and the goal of integrating all environmental data within a single system.

Third, universalizing ambitions are further limited by how technologies simulate biodiversity data by separating its entities and only collecting data on the absence or presence of predetermined species. By neglecting how biodiversity indicators are also present in species relations, local knowledge, and their entanglements, new digital infrastructures further fragment ecosystems into separately measurable entities.

Fourth, despite frequently articulated aims of participatory biodiversity monitoring initiatives, many of the projects involved in this study saw their technological ambitions limiting the involvement of less powerful stakeholders. Here, typical users lack the advanced technological skills or interest to engage with advertised open-source and open-data features. Participants also highlighted that the pace at which new digital technologies are proposed, funded, and advanced makes it challenging to critically interfere in these developments.

These digital logics illustrate how the future of biodiversity monitoring will be influenced through the formations and structures that are set out in digital innovation processes. Technologists and funders thereby are powerful stakeholders who are shaping biodiversity futures, which become driven by digital innovation for its own sake. Digital twin technologies will potentially play an important role in preventing further biodiversity loss and restoring ecosystems. However, they are limited by structures that direct biodiversity knowledge towards certain areas while neglecting others. In order to better encompass multidimensional biodiversity processes, recognize partial knowledge, and monitor ecosystems more relationally, the digital processes summarized in this article must be rethought.

The new digital logics of biodiversity knowledge can be partly addressed. First, funders can demand more diverse teams, who can continuously question what directs technological decisions and how monitoring practices are determined and funded. Second, attending to biodiversity knowledge that is not captured by technology can involve recognizing data absences, local tensions, land-use conflicts, and the socio-cultural aspects of local biodiversity. Third, while the inherent limitations in producing complex biodiversity knowledge cannot be eliminated, they should be deliberately included in the design of new monitoring technologies. Rather than aiming to create complete copies or twins of environmental phenomena, technologists must deliberately acknowledge and identify their partial representations. Fourth, digital innovation must move beyond a focus on only identifying individual species and engage with the many other ways in which biodiversity becomes apparent. This includes valuing incoherent data, outliers, unique relations between species, and ecosystem processes. Last, as technological processes become increasingly complex, fast-paced, and data-intensive, efforts towards public participation and democratic decision-making must become more integrated parts of developmental processes. Instead of hiding behind algorithms and complexity, emerging biodiversity technologies must involve meaningful public engagement by enabling people to understand, engage in critique of, and contribute to their digital operations.
